# Clinicians’ and healthcare leaders’ perspectives on suitability of virtual healthcare in metropolitan versus rural areas

**DOI:** 10.1038/s44401-024-00007-y

**Published:** 2025-03-01

**Authors:** K. McGrath, C. Grogan, A. Barron, I. Smith, S. Kanagarajah, S.-E. Smith, O. J. Fisher

**Affiliations:** 1 Wesley Research Institute, Brisbane, Australia; 2 Faculty of Health, University of Queensland, Brisbane, Australia; 3 UnitingCare Queensland, Brisbane, Australia; 4 St. Andrew’s War Memorial Hospital, Brisbane, Australia; 5 Queensland Physician Care, Brisbane, Australia; 6 BlueCare, Caboolture, Australia; 7 Charles Darwin University, Darwin, Australia

**Keywords:** Health policy, Health services

## Abstract

Australia’s geographically tiered health system is reinforced by long-term urban-centric policymaking. People in rural and remote areas have poor health outcomes, high preventable hospitalisations, and premature death. Virtual healthcare can improve access, but many clinicians and health service leaders (leaders) consider it substandard to face-to-face. This qualitative thematic study interrogated hidden meanings representing unconscious bias. Interviews were conducted with clinicians and leaders (February–July 2023, Queensland, Australia) to inform the design/delivery of a private virtual hospital. 26 participants: 5 leaders, 3 clinicians, 18 both leaders and clinicians. Three themes: (1) traditional face-to-face healthcare is better than telehealth; (2) virtual healthcare offers an opportunity for rural and remote residents with limited access to face-to-face healthcare; and (3) telehealth is better than nothing. Taken together, the themes indicate tacit acceptance of a lower healthcare standard for people in rural and remote areas. Acceptance of a lower standard may unconsciously negatively influence healthcare service design.

## Introduction

People living in rural and remote Australia deserve equivalent healthcare to those in urban areas. Instead, Australia has a geographically tiered health system and a lack of healthcare equity^1–5^. Many rural and remote communities have limited or no access to timely, clinically and culturally appropriate health services leading to poor health outcomes and quality of life, preventable hospitalisations, and early death^1,2,5^. It is well documented that healthcare barriers in rural areas differ greatly from those in urban areas, and these needs are not homogenous across rural and remote communities^2,3,6,7^. Insufficient progress has been made in addressing this complex issue^1,2,5^. Healthcare innovation is limited by restrictive and inflexible funding structures^4,8^. Australia has a long history of urban-centric paternalism in healthcare policymaking that prioritises people in metropolitan areas^3^. Likewise, clinicians and healthcare decision-makers are known to have conscious and unconscious biases that impact the design and delivery of health services^9,10^. Understanding and challenging urban-centric biases is an important step towards ending geographical healthcare inequity. Indeed, addressing and compensating for bias is a critical responsibility of clinicians, healthcare decision-makers and policymakers, and doing so requires insight and understanding of the bias and its potential impacts^11^.

Unless otherwise specified, throughout this study, we have used the modified Monash model of relative remoteness^12^, which is a seven-point scale from MM1—metropolitan areas/major cities, to MM7 very remote communities. MM2 is referred to here as “regional”, MM3–5 are referred to collectively as “rural” areas, and MM6 and 7 are referred to collectively as “remote” areas. Approximately 28% of the Australian population live outside of major cities in regional, rural or remote areas^2,13^. There are often vast distances to travel to access basic services and fewer health, employment and education options^2^. The median age at death in Australian major cities is 80 and 85 years for males and females, respectively, reducing with remoteness level to just 67 and 69 years, respectively, in very remote areas^2^. There is a significant healthcare expenditure gap of $848.02 per capita between people living in metropolitan and non-metropolitan areas ($7103.72 versus $6255.70, respectively, financial year 2020–21)^4^. General practice attendances in metropolitan areas are 6.6 per person per year but only 4.2 in remote and 3.4 in very remote communities, indicating a lack of preventative primary care^2^. This is evidenced by routine screening rates such as for bowel, breast and cervical cancer declining with remoteness, leading to a high rate of potentially preventable deaths, which rises starkly with remoteness level^2^.

Virtual healthcare services have been proposed as a strategy for improving geographical healthcare equity by providing increased and timely access to primary care, medical specialists, and allied health services^3,5^. Although there is considerable evidence that virtual healthcare and virtual hospitals can deliver comparable health outcomes to traditional face-to-face services (e.g.^14–16^), many clinicians remain sceptical that an acceptable standard of care can be delivered through virtual models^17–19^. It is, therefore, imperative to ensure that Australia’s current geographically tiered health system is not reinforced through the implementation of substandard healthcare models. There is more to virtual healthcare than just telehealth. Many hybrid models have been found to be both clinically effective and safe. Although telehealth is an important and necessary component, these hybrid models pair telehealth consultation (e.g., medical specialist), with a face-to-face clinician such as a nurse who visits the home or community, and/or remote monitoring devices (e.g.^15,16,20–22^). For the purposes of this study, we have taken a broad definition of virtual healthcare as digitally enabled healthcare activity supported by information and communication technology services. This includes but is not limited to telehealth-only services, hospital-in-the-home models, virtual wards, and virtual emergency departments. We aimed to identify clinicians’ and healthcare leaders’ perspectives on the suitability of virtual healthcare for people living in metropolitan versus rural and remote Queensland.

## Methods

This qualitative study was conducted prior to the implementation of a private, not-for-profit virtual hospital in Queensland, Australia. It was part of a broader programme of research to inform the design and implementation of a virtual hospital in both metropolitan and non-metropolitan areas by identifying contextual barriers, enablers and considerations^8^ and to co-design a set of principles and inform the long-term vision of a virtual hospital^23^. The data utilised in this case study was collected via a series of context assessment interviews conducted between February and July 2023. For the purposes of this study, we have referred to all participants who had a leadership role, such as a management role in a hospital or aged care service, as a health service leader.

### Research question

What are the perceptions of clinicians and healthcare decision-makers about the suitability of virtual healthcare for people living in metropolitan versus rural and remote Australia?

### Participants and recruitment

Detailed methods, including the implementation science approach used and interview guides, are available elsewhere^8^. Participants in the semi-structured interviews were invited based on their work roles. Sample size was based on the representation of key stakeholder groups rather than data saturation. Stakeholder groups were: health and aged care service leaders (referred to hereafter as health service leaders), and health and aged care workers including doctors, nurses, allied health practitioners, and personal care workers (hereafter referred to collectively as clinicians). These were broad groups of participants with diverse backgrounds that represent two distinct groups: leadership and decision-making roles and frontline or patient-focused roles. Purposeful snowball sampling was used^24^. Interviews were conducted online via Teams or Zoom or in person and were audio recorded and transcribed by a professional transcription service.

### Scope

This study focused solely on the perceptions of clinicians and health service leaders relating to the suitability of virtual healthcare in rural and remote versus metropolitan areas. Although interviews were conducted with health consumers and informal carers as part of the broader research programme, these interviews were excluded from this analysis because these data did not address the research question.

### Analysis

The study of bias, particularly unconscious bias, is complex but important. Identifying unconscious bias requires the interrogation of both explicit statements and implicit, hidden meanings. The authors acknowledge the inherent subjectivity of examining hidden meanings in qualitative data^25^. There is no agreement in the literature on the best method for identifying hidden meaning, but there are techniques that can maximise the reliability of analysis and interpretation, such as, including interviewers’ recollections of interview dynamics and non-verbal cues, using multiple coders and reflective practices^25,26^. Having multiple coders regularly meet to discuss and unpack the trustworthiness of interpretations and scrutinising the relationship between data and interpretations validated that any claims are grounded in the data.

In this study, we have endeavoured to be both transparent and reflexive in our methods. All data has been analysed at least three times. Initial qualitative analyses were conducted as described in Fisher et al. ^8^ using a framework analysis method and the planning and evaluating remote consultation services (PERCS) framework. It was noted by both interviewers who conducted the initial coding that an important theme across the clinician and health service leader data was the differences in participants’ perceptions of the suitability of virtual healthcare for people living in metropolitan versus rural and remote areas.

A second analysis was then conducted. All clinician and health service leader transcripts were searched for text relating to non-metropolitan areas using the search terms: “rural”, “remote”, and “regional”. All quotes relating to virtual healthcare in rural and remote areas that were identified in this initial scan were analysed using an inductive thematic analysis approach^27,28^ to develop initial themes. These initial themes and relevant quotes were discussed and agreed upon between the three coders.

All complete transcripts were then reviewed a third time by one or more coders to identify whether each individual participant agreed, disagreed, or did not mention each theme. Both explicit and implied statements that represented a theme were included. For example, if a participant stated that virtual healthcare could address service gaps in rural and remote areas, this was considered an explicit agreement with the theme “virtual healthcare offers an opportunity for rural and remote residents with limited access to face-to-face healthcare.” If a participant stated that they could see a use case for telehealth but only as a complement to, not a replacement for, face-to-face healthcare delivery, this was coded as implicit agreement with the theme “traditional face-to-face healthcare is better than telehealth”. Agreement/disagreement/lack of discussion of each theme was recorded for each participant, along with the rationale for the coder’s decision. The rationales for coding decisions were then discussed as a coding team to ensure consistency in interpretation. Disagreements in interpretation were discussed between the coders. Interviewers’ recollections of participants’ tone, body language, and emphasis were discussed where there was disagreement in interpretation. Where necessary, the transcripts were reviewed by all three coders so a consensus decision could be reached.

### Ethical approval

Ethical approval was received on 9 January 2023 by the UnitingCare Queensland Human Research Ethics Committee, Reference: Fisher_20221207. Participation was by informed consent. This research was conducted in accordance with the relevant Australian research guidelines and regulations, including the National Statement on Ethical Conduct in Human Research. This research was conducted in accordance with the Declaration of Helsinki.

## Results

### Participants

Of the 37 interview participants, 11 were either a consumer, carer or both, with no clinical or health service leadership role. These 11 participants were therefore excluded from this study. Characteristics of the remaining 26 participants are outlined in Table 1.

**Table 1:** Interview participants

		*n* (%)
Role^a^	Health Service Leader	23 (52.3%)
	Nurse	11 (25.0%)
	Doctor	6 (13.6%)
	Allied Health	2 (4.5%)
	Pharmacy	1 (2.3%)
	Personal Carer	1 (2.3%)
	Total	44 (100%)
Location	Metropolitan	22 (84.6%)
	Regional	2 (7.7%)
	Rural and Remote	2 (7.7%)
	Total	26 (100%)
Sex	Male	9 (34.6%)
	Female	17 (65.4%)
	Total	26 (100%)

^a^Some participants held more than one role.

### Themes

Three overall themes were present in the data: (1) traditional face-to-face healthcare is better than telehealth; (2) virtual healthcare offers an opportunity for rural and remote residents with limited access to face-to-face healthcare; (3) telehealth is better than nothing. Overall, 23 (88.5%) of the 26 participants discussed at least one of the themes, and 12 (46.2.0%) expressed views that agreed with all three themes (Table 2). All participants who discussed themes two and three expressed views that agreed with the theme. For theme one, there was some disagreement: three (13.6%) of the participants who discussed whether face-to-face is better than virtual healthcare (*n* = 22, 84.6% of all participants) disagreed with the theme. All participants who disagreed with theme one were in leadership roles and had a nursing background. There were no differences in responses based on participants’ geographical locations or roles.

**Table 2:** Participants’ agreement and disagreement with themes

Participant number—new for publication	Role/s	Locality	Theme one	Theme two	Theme three
CL1	Leader	Metro	Agree	Not discussed	Not discussed
CL2	Leader, Nurse	Metro	Not discussed	Not discussed	Agree
CL3	Leader, Nurse	Metro	Disagree	Not discussed	Not discussed
CL4	Leader, Nurse	Non-metro	Agree	Agree	Agree
CL5	Doctor	Metro	Agree	Agree	Agree
CL6	Leader, Other	Metro	Agree	Agree	Agree
CL7	Leader, Doctor	Metro	Agree	Agree	Agree
CL8	Leader, Nurse	Metro	Agree	Agree	Agree
CL9	Leader	Metro	Not discussed	Not discussed	Not discussed
CL10	Leader, Doctor	Metro	Agree	Agree	Agree
CL11	Leader	Metro	Agree	Not discussed	Not discussed
CL12	Leader, Nurse	Metro	Agree	Agree	Agree
CL13	Leader, Other	Metro	Agree	Not discussed	Agree
CL14	Leader, Nurse	Metro	Disagree	Not discussed	Not discussed
CL15	Leader, Nurse	Metro	Not discussed	Not discussed	Not discussed
CL16	Other	Metro	Agree	Not discussed	Agree
CL17	Nurse	Metro	Not discussed	Not discussed	Not discussed
CL18	Leader, Other	Metro	Agree	Agree	Agree
CL19	Leader, Nurse	Non-metro	Agree	Agree	Agree
CL20	Leader	Non-metro	Agree	Not discussed	Not discussed
CL21	Leader, Doctor	Metro	Agree	Agree	Agree
CL22	Leader, Doctor	Metro	Agree	Not discussed	Agree
CL23	Leader	Metro	Agree	Agree	Agree
CL24	Leader, Nurse	Metro	Disagree	Not discussed	Agree
CL25	Leader, Doctor	Metro	Agree	Agree	Agree
CL26	Leader, Nurse	Non-metro	Disagree	Not discussed	Not discussed

Metro = major city, Non-metro = regional, rural or remote area, Other = pharmacists, allied health practitioners and personal carers.

### Theme one: traditional face-to-face healthcare is better than telehealth

The majority of participants expressed a view that virtual healthcare, particularly telehealth, is a substandard alternative to traditional face-to-face healthcare delivery. Some discussed the potential for hybrid models, with a person such as a nurse or personal carer face-to-face with a consumer and a doctor via telehealth or the use of monitoring devices. However, the strong underlying sentiment from the majority (*n* = 18, 69.2%) was that virtual healthcare is not comparable in quality with face-to-face. This was not a consistent theme for all participants— three participants reported they did not consider F2F to be better than telehealth or virtual healthcare: “I’m actually an advocate that don’t bring people in hospitals because we do harm when people come into hospitals.” CL26, nurse and health service leader. The following subthemes were noted:

Subtheme (a) People in affluent metropolitan areas get the best healthcare available: There are more provider options in affluent areas of major cities, and consumers who have the capacity to pay a gap fee are able to choose their preferred providers.

“…if you’re on the Northern Beaches of Sydney and of course… you want your GP that’s been looking after you for all of your life… and you’re happy to pay 100 bucks over the Medicare… Well, of course, that’s great ideologically for you to have that person that you know… So… I think… relying on this model of personal choice is fine in the northern beaches, in the affluent areas, but most of the time the whole thing fails.” CL25, doctor and health service leader

Subtheme (b) Need for healing touch: Some participants stated that the importance of face-to-face care goes beyond simply the ability to conduct a clinical assessment. They expressed that the healing touch between a practitioner and consumer is an important component of a medical consultation.

“I think we underestimate the value of both clinical examination and the value of the healing touch or the hand on the shoulder, the eye contact, the being in the room and feeling the unsaid, feeling of the nurse, you know, the mother who’s just out of eye contact that you can’t see distressed.” CL7, doctor

Subtheme c) Telehealth is risky: Some participants raised substantial concerns about the risks of conducting a medical assessment via virtual healthcare, even with a person such as a nurse face-to-face with the patient. Examples were provided of misdiagnoses, incomplete assessments, and communication challenges.

“I can catalogue just over the last month, I’ve seen errors in telehealth… They’ve missed pneumonia, they’ve missed kidney stones, they’ve missed a bowel obstruction, they’ve missed an ischaemic valve, they’ve missed an MI [myocardial infarction]. You know, all because they think they can do all these things on telehealth, because they’ve not partnered it with a very clear idea of, well, these are the things I can do on telehealth and these are the things that an older person needs to have done in person, in a sense, a proper hands on approach to that person.” CL21, doctor

“It’s not an easy thing to do over the phone, by any stretch. I’ve done it, it’s not easy, it’s not even easy getting in a car and going out to see someone in a non-familiar environment… the data you need to make a diagnosis or to ensure a complete assessment, I think, will be really difficult in a virtual environment, ‘cause you need to have on the other end, someone that can provide you with accurate information.” CL12, nurse and health service leader

Subtheme d) Telehealth can be a useful complement but not a substitute: There was strong agreement amongst participants that telehealth alone is not sufficient for high-quality healthcare delivery. This was seen as particularly important for Aboriginal and Torres Strait Islander peoples but was a consistent message across all patient groups. The need for clear communication between the clinician and consumer was a key point raised.

“Having Indigenous clients, so with telehealth, we’ve got to think seriously about communication. You know, you need to, you need to be able to see the body of a person who’s expressing their desire to look after you. To them you are reading their body, not just hearing their words. When you come from a custom that is, or a culture which is a verbal culture, not a written culture. You know. So, you’re reading everything about what that practitioner is actually saying to you. And it’s really interesting, because some of the old grannies, they’ll look up. Like shy. But what they’re doing is they’re looking for your eye movements related to what you’re telling them. Really, it’s really interesting to watch. Culture in practice. So anyway. So telemedicine in Indigenous environments has not had a lot of success, to my way of thinking.” CL19, nurse and health service leader

### Theme two: virtual healthcare offers an opportunity for rural and remote residents with limited access to face-to-face healthcare

There was consistent and strong agreement with this theme by the 12 people (46.2%) who discussed healthcare access in rural and remote areas. Participants described barriers to access, including a lack of primary care, specialist medical and allied health providers; long travel distances to access routine and emergency medical care; and long waiting lists.

“…we run a lot of aged care facilities across the country, some with no access to medical care at all, and I mean that quite literally, and so this provides them with a potential opportunity to get medical care that they may not otherwise get.”—CL24, health service leader

“I think clearly outside of South East Queensland there’s, you know, inadequate access to high level emergency facilities, intensive care, paediatrics, obstetrics, so all sorts of things that probably elements of it could be delivered virtually.”—CL10, doctor and health service leader

“If you live in Hervey Bay, which has the oldest demographic in Queensland, there’s no geriatrician. There’s no dementia diagnosis in Hervey Bay.” CL7, doctor

“You know some places you may have, they might come in once a month, they may come in twice, you know every second month but they’ve got a humungous list of who they need to see… Like even a skin specialist would be absolutely filled up and then, you’ve missed out.” CL4, nurse and health service leader

Expedience measures to address this limited access to face-to-face healthcare in rural and remote areas were described, such as employing nurse practitioners with a broad remit. However, these expedience measures are merely a stop-gap, and cannot adequately address the health needs of these communities. “Nurse practitioners… provide a lot of our indigenous health in remote communities because no one else wants to or has to, but they will go, and they flow into the cracks where service is poor because they’re motivated differently.” CL23, health service leader

### Theme three: virtual healthcare is better than nothing

If you cannot get face-to-face care, virtual healthcare is considered better than no care at all. Virtual healthcare was raised as a potential stop-gap to address staff shortages. Most of the examples participants gave about potential use-cases for virtual healthcare involved a consumer not being able to access face-to-face healthcare and using virtual as a second-best option.

“…where I’ve seen it work elsewhere, they’re very different communities. They’re a lot more rural and remote. You know, ICUs managed remotely and things like that, where you can’t get staff.”— CL10, nurse and health service leader

“…and if someone chops their leg off or does something, you know, thinks they’re having a heart attack or whatever problem you might have in far western Queensland, if you’re, you know, 200, 300 Ks from Longreach or Winton on a station and you phone the RFDS [Royal Flying Doctor’s Service] and they’re like well these are the things you need to do. Obviously that’s not best practice, and if you’re in a city setting, you can come to an ED or you go to a hospital, you but, you can certainly manage a lot, and I guess it’s like necessity is the mother of invention, you know, there’s no other choice is there, so you just do it that way.” CL23, health service leader

“But we would use it for sure. I mean especially those days when we get, you know, which is becoming more and more common where the hospital rings down there’s no beds. There’s no beds and you’re like, well, what do I do now I’ve got ten people here and nowhere to send them. If I could send two of them home with some sort of confident plan then that’s, we’re happy with that.” CL22, doctor and health service leader

“And, and they pushed me [telehealth trolley] to the edge of the bed and then I would do the assessment remotely. Was that perfect, perfect, 100 per cent as good as what you get in my clinic? No, but it was pretty jolly good.”—CL7, doctor

## Discussion

People living in rural and remote areas deserve healthcare equivalent to those in metropolitan areas, but this is not their reality. Two key issues were highlighted in this study: 1. Clinicians’ and healthcare decision-makers’ attitudes towards virtual healthcare do not align with research evidence on the safety and clinical effectiveness of virtual healthcare; and 2. Clinicians’ and healthcare decision-makers’ attitudes towards the suitability of virtual healthcare differed based on whether a consumer lives in a rural, remote or metropolitan setting. It was seen as acceptable for consumers living in rural and remote areas, but an inferior substitute for consumers in metropolitan areas who have ready access to traditional face-to-face services. This was consistent regardless of where a participant was located. Three themes were identified: “traditional face-to-face healthcare is better than telehealth,” “virtual healthcare offers an opportunity for rural and remote residents with limited access to face-to-face healthcare,” and “telehealth is better than nothing.”

There is extensive evidence on the safety and effectiveness of virtual hospitals, including virtual wards^15,21^ and hospitals in the home^14,29^. The authors acknowledge that to date the bulk of this evidence has focused on hospital in the home models where a nurse attends a patient in their home to provide face-to-face care with medical consultation via telehealth, and COVID-19 virtual wards. However, the evidence base for other clinical conditions and models is quickly building. While there is currently no technology readily available to conduct some types of medical care, such as orthopaedic surgery, for specialities where virtual healthcare options are available, the evidence demonstrates equivalent clinical outcomes compared with traditional face-to-face healthcare delivery^14,15^. Interestingly, in this study, the majority of clinicians and health service leaders expressed the view that virtual healthcare is substandard to face-to-face, which does not align with the most up-to-date evidence. It is notable, however, that four clinicians reported that virtual healthcare can be superior to face-to-face care in a hospital, e.g., because of decreased risks of some types of hospital-acquired complications such as hospital-acquired infection.

Taken together, the three themes indicated a tacit acceptance of a lower standard of healthcare for people in rural and remote areas who otherwise have little to no healthcare access. Clinicians and health service leaders inferred a different calculation of risk and acceptability based on whether a consumer had other face-to-face healthcare options. Virtual healthcare was considered substandard for consumers in metropolitan areas, particularly in affluent areas, who can readily access their choice of face-to-face providers. This indicates an unconscious urban-centric bias aligning with the long-standing urban paternalism and blind spots demonstrated by Australian healthcare policymakers and decision makers^3,30,31^. Although it was not explicitly stated by any participant, the implication remains that if a person does not have ready access to face-to-face healthcare, a lower standard of healthcare will suffice.

From a human rights perspective, healthcare that is ‘better than nothing’ is not good enough. The United Nations’ Sustainable Development Goal 3 is to “ensure healthy lives and promote well-being for all at all ages.”^32^ Inadequate progress has been made towards meeting this goal for people living in rural and remote Australia, and Malekpour et al. ^33^ argue that scientists must identify impediments that are preventing its attainment. The authors contend that Australia’s ongoing failure to effectively address geographical healthcare inequities has, in part, resulted from tacit acceptance by clinicians, healthcare decision-makers and policymakers of substandard healthcare services and access outside of metropolitan areas. The significant challenge to deliver equitable healthcare to a decentralised, geographically dispersed population with limited resources is considered too difficult, and this leads to the acceptance of a lower standard of care. Unaddressed, this bias has the potential to continue to negatively influence healthcare service planning, funding and delivery in rural and remote Australia, reinforcing the current geographically tiered health system^1,2,4^ and preventing parity of health outcomes for rural and remote Australians.

Past policy decisions, such as the example outlined by Caffery et al. ^3^ of the cessation of Medicare item numbers for telehealth services post-COVID-19 pandemic, have disproportionately impacted people in rural and remote areas. During the COVID-19 pandemic people living in rural and remote areas were temporarily able to access general practitioners and other healthcare providers from outside of their local area via telehealth, subsidised by Australia’s Medicare programme. This substantially impacted people in rural and remote areas during this period by improving equitable access to timely and appropriate medical care from a provider outside of their local area. Rolling out models of care that may be effective in metropolitan settings without adaptation for rural and remote areas is also unsuitable. One example is the proposal to improve access for people in regional, rural and remote areas to better cancer care via Comprehensive Cancer Centres, which Sabesan et al. ^34^ argue is based on metropolitan assumptions and will not meet the needs of rural and remote communities. Opening a large medical service in a regional area is not enough to improve access to care for people in rural and remote areas who may still need to drive multiple days and take extensive time away from their homes, families, jobs and communities to access these Comprehensive Cancer Centres. Without providing a networked and holistic approach that takes into account the heterogeneity of rural and remote areas, a widening of health inequalities between rural, remote and metropolitan settings is likely to occur. Understanding and addressing this bias is timely and necessary as virtual hospitals and telehealth services are rapidly being implemented internationally^8,14,15,20^. These services must be designed and delivered to the same standard in rural and remote areas as would be considered acceptable in major cities. One successful example of a locally based rural and remote service which improves access to tele-mental health is Isaac Navicare in the Bowen Basin region of rural and remote Queensland^7^. Local care navigators based within the remote area support people seeking mental health, alcohol and other drugs, suicide prevention and other wraparound services such as homelessness and domestic violence services. Coupling local supportive care navigators with telehealth providers from across Australia increased timely access to and uptake of telehealth mental health services.

Bias can be addressed, but it must first be identified and acknowledged. Urban-centric biases of clinicians, health service leaders and policymakers can and have led to devastating consequences for people living in rural and remote areas, such as substandard health outcomes, poor quality of life, and early death. In this study, we examined clinicians’ and health service leaders’ attitudes towards the suitability of virtual healthcare for metropolitan versus rural and remote healthcare delivery. An implicit urban-centric bias was identified. Participants described virtual healthcare as substandard compared with face-to-face healthcare, but for people in rural and remote areas it presented an opportunity to access some level of healthcare and was therefore considered acceptable given the lack of alternative options. The authors contend that without acknowledging and addressing this tacit acceptance of a potentially lower standard of care for people in rural and remote areas, this bias may unconsciously negatively impact the design and delivery of virtual healthcare services for people in rural and remote areas. Clinicians and healthcare decision-makers may thus accept a lower standard of care for people in rural and remote areas than would be considered acceptable in metropolitan areas. This would thus reinforce Australia’s geographically tiered health system and perpetuate health inequities for rural and remote residents.

### Limitations

The study of hidden meaning and unconscious bias is complex and requires the research team to participate in regular reflection and unpacking of their own perspectives. Although robust and transparent methods have been used to ensure that the results are authentic and reliable, a possibility remains that the research team have misinterpreted the participants’ meaning. Further research in other settings is important to understand whether these results are reflected in a broader population and whether these perspectives may change over time.

## Data Availability

The data for this study will not be shared as it is potentially identifiable, and we do not have permission from the participants or ethics approval to do so. Transcripts clearly identify the roles and responsibilities of each participant throughout, both directly and indirectly, and are not able to be de-identified. The coding dictionary is available at the request of the corresponding author.

## Reflexivity statement

The research team consisted of career researchers with relevant qualifications and experience who are employed by the research partner (O.J.F., K.M., C.G.), a Ph.D. qualified career researcher employed by the industry partner (IS), employees of the industry partner with a leadership role in design and implementation of the virtual hospital (A.B., S.-E.S.), and an independent medical practitioner who volunteered their time to provide relevant clinical input into the study design (S.K.). A.B. also had a leadership role at the research partner. O.J.F., K.M., C.G., A.B., S.K. and I.S. had no supervisory relationship with any participant. S.-E.S. works in a statewide leadership role and, therefore, has a leadership relationship with some participants. A.B., I.S., S.K. and S.-E.S. did not obtain participants’ consent to participate, conduct interviews or review unredacted data. At the time of the study O.J.F., C.G., A.B., S.K. and I.S. all worked in a major city. S.-E.S. worked in a regional city. K.M. worked in a small remote township. K.M., C.G. and O.J.F. conducted the majority of the analysis and interpretation of these data. O.J.F. is a Ph.D.-qualified implementation scientist and health services researcher. O.J.F. is a former occupational therapist and now has a full-time Senior Research Fellow role leading a programme of virtual healthcare research. O.J.F. has previously lived and worked as a clinician in both rural and remote and metropolitan Australia. O.J.F. had previously met some participants through their research role but had no supervisory relationship with any participant. O.J.F. has experience in qualitative research, including implementation science and thematic analysis methods. C.G. is a Ph.D.-qualified social worker with extensive experience in the care of older persons. C.G. is an aged care and health services Research Fellow with expertise in qualitative research and a background in social work. C.G. had no known prior relationships with any participants that C.G. interviewed. C.G. had met some participants that they did not interview prior to the study through their research role. C.G. has not lived or worked in rural and remote areas. At the time of the study, K.M. was employed by the research partner as a Research Assistant and rural and remote mental health Care Navigator (non-research role). K.M. has a background in biological sciences and health services research. K.M. lives in remote Queensland.

## EQUATOR guideline

This manuscript complies with the relevant EQUATOR reporting guideline: the Standards for Reporting Qualitative Research.
